# Epicutaneous allergen application preferentially boosts specific T cell responses in sensitized patients

**DOI:** 10.1038/s41598-017-10278-1

**Published:** 2017-09-14

**Authors:** Raffaela Campana, Katharina Moritz, Angela Neubauer, Hans Huber, Rainer Henning, Tess M. Brodie, Alexandra Kaider, Federica Sallusto, Stefan Wöhrl, Rudolf Valenta

**Affiliations:** 1 Division of Immunopathology, Department of Pathophysiology and Allergy Research, Center of Pathophysiology, Infectiology and Immunology, Vienna General Hospital (AKH), Medical University of Vienna, Vienna, Austria; 2 Division of Immunology, Allergy and Infectious Diseases (DIAID), Department of Dermatology, Vienna General Hospital (AKH), Medical University of Vienna, Vienna, Austria; 3 Biomay AG, Vienna Competence Center, Vienna, Austria; 4 Cellular Immunology Laboratory, Institute for Research in Biomedicine, Bellinzona, Switzerland; 5 Center for Medical Statistics, Informatics and Intelligent Systems, Section for Clinical Biometrics, Medical University of Vienna, Vienna, Austria; 6 Institute of Microbiology, ETH Zuerich, Switzerland

## Abstract

The effects of epicutaneous allergen administration on systemic immune responses in allergic and non-allergic individuals has not been investigated with defined allergen molecules. We studied the effects of epicutaneous administration of rBet v 1 and rBet v 1 fragments on systemic immune responses in allergic and non-allergic subjects. We conducted a clinical trial in which rBet v 1 and two hypoallergenic rBet v 1 fragments were applied epicutaneously by atopy patch testing (APT) to 15 birch pollen (bp) allergic patients suffering from atopic dermatitis, 5 bp-allergic patients suffering from rhinoconjunctivitis only, 5 patients with respiratory allergy without bp allergy and 5 non-allergic individuals. Epicutaneous administration of rBet v 1 and rBet v 1 fragments led to strong and significant increases of allergen-specific T cell proliferation (CLA+ and CCR4+T cell responses) only in bp-allergic patients with a positive APT reaction. There were no relevant changes of Bet v 1-specific IgE and IgG responses. No changes were noted in allergic subjects without bp allergy and in non-allergic subjects. Epicutaneous allergen application boosts specific T cell but not antibody responses mainly in allergic, APT-positive patients suggesting IgE-facilitated allergen presentation as mechanism for its effects on systemic allergen-specific immune responses.

## Introduction

Epicutaneous allergen administration in the form of atopy patch testing (APT) has been widely used to reveal T cell-mediated late phase allergic inflammation in patients suffering from atopic dermatitis^1^. However, T cell-mediated allergic inflammation has also gained considerable attention as a possible mechanism for late phase side effects in the course of allergen-specific immunotherapy (AIT)^2^. An elegant study has shown that the administration of T cell epitope-containing peptides without IgE reactivity could induce T cell-dependent MHC-restricted late phase respiratory symptoms^3^. We therefore investigated if late-phase side effects observed in the course of AIT with recombinant non-IgE-reactive, T cell epitope-containing derivatives of the major birch pollen allergen Bet v 1 may be also caused by a T cell-dependent and IgE-independent mechanism^4^. In fact, it could be shown by APT that non-IgE-reactive Bet v 1 derivatives induced T cell mediated late phase skin inflammation in atopic dermatitis (AD) patients^5^. Recently we could show in a clinical trial that such late phase skin reactions could be also induced in patients suffering only from birch pollen-induced respiratory symptoms without AD^6^. Moreover we also showed that such reactions are very common and thus may explain the frequent occurrence of late phase reactions observed during AIT with T cell epitope-containing allergen derivatives (i.e., recombinant hypoallergens, allergoids)^6^.

However the effects of epicutaneous allergen administration on allergen-specific systemic immune responses has not been studied in detail. In particular there are no data regarding the effects of epicutaneous administration of recombinant allergens in well characterized allergic patients and non-allergic control subjects. So far only the effects of intranasal administration of recombinant allergens and hypoallergenic allergen derivatives on systemic immune responses in allergic patients have been investigated^7, 8^. These studies showed that a single intranasal application of folded, IgE-reactive Bet v 1 allergen, but not of hypoallergenic Bet v 1 derivatives, induced strong boosts of systemic IgE responses 6 weeks after intranasal administration.

Here, we investigated the effects of epicutaneous application of the IgE-reactive recombinant form of the major birch pollen allergen, rBet v 1 (aa: 1–160) and two recombinant hypoallergenic rBet v 1 fragments (F1: aa 1–74, F2: aa 75–160)^9^ on systemic allergen-specific antibody, T cell and cytokine responses.

## Results

### Study design

**Figure 1:**
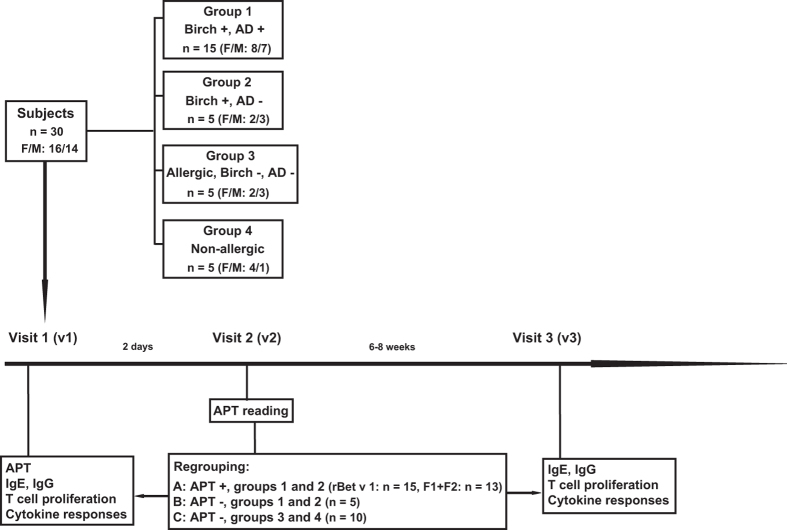
Study design. Thirty subjects, 15 birch pollen allergic patients suffering from AD (group 1), 5 birch pollen-related rhinoconjunctivitis patients without AD (group 2), 5 allergic patients without birch pollen allergy and without AD (group 3), and 5 non-allergic individuals (group 4) were subjected to APT, analysis of allergen-specific antibody, T cell and cytokine responses as well as SPT at visit 1. APT reading was done after 2 days (visit 2), and allergen-specific antibody, T cell and cytokine responses were analysed again 6–8 weeks after APT application (visit 3). After APT reading subjects were regrouped in A (APT-positive birch pollen allergic patients: rBet v 1, n = 15; F1 + F2, n = 13), B (APT-negative birch pollen allergic patients: n = 5) and C (APT-negative subjects without birch pollen allergy: n = 10).

Here we investigated the effects of epicutaneous allergen application on systemic cellular, humoral and cytokine responses as a follow-up to an earlier study. In the earlier study, subjects (n = 30, 16 female and 14 male; group 1, birch pollen allergic patients with AD exacerbations upon exposure to birch pollen (n = 15), group 2, birch pollen-related rhinoconjunctivitis patients without AD (n = 5), group 3, patients with only respiratory forms of allergy to allergen sources other than birch pollen (n = 5) and group 4, non-allergic individuals (n = 5))^6^ were subjected to atopy patch testing with rBet v 1 and hypoallergenic T cell epitope-containing rBet v 1 fragments (Fig. 1, visit 1) to identify immunological surrogate markers for a positive patch test reaction.

For this follow-up study, subjects were regrouped according to the development of positive or negative patch reactions at visit 2 (i.e., 2 days after APT) (Fig. 1) and the presence or absence of birch pollen allergy into three groups:

Group A: positive APT reaction and birch pollen allergy (n = 15: Eleven patients from group 1 and 4 patients from group 2).

Group B: negative APT reaction and birch pollen allergy (n = 5: Four patients from group 1 and one patient from group 2).

Group C: negative APT reaction without birch pollen allergy (n = 10: Five from group 3 and five from group 4) (Fig. 1). Subjects from group C had no detectable Bet v 1-specific IgE levels in their serum (data not shown).

The goal of this study was to compare IgE, IgG (IgG subclass), T cell and cytokine responses specific for rBet v 1 and the hypoallergenic, T cell epitope-containing rBet v 1 fragments before APT (visit 1) and 6–8 weeks after APT at visit 3 (Fig. 1).

### Epicutaneous application of Bet v 1 and hypoallergenic Bet v 1 fragments does not induce increases of allergen-specific serum IgE antibody levels

**Figure 2:**
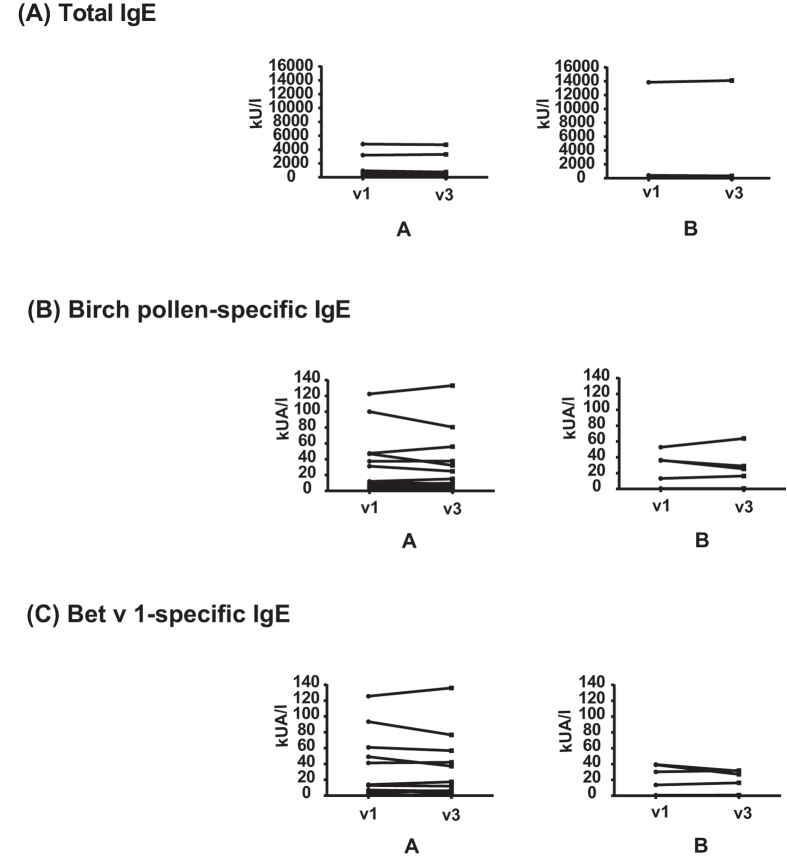
Total IgE and allergen-specific IgE levels before and after APT. On the y-axes total IgE **(A)**, birch pollen-specific IgE **(B)** and rBet v 1-specific IgE levels **(C)** before (v1) and 6–8 weeks after APT application (v3) are shown for A (APT-positive birch pollen allergic patients) and B (APT-negative birch pollen allergic patients).

In a first set of experiments, total IgE and specific-IgE antibody levels against natural birch pollen as well as against rBet v 1 were determined in the birch pollen allergic patients (group A: APT-positive birch pollen allergic patients, group B: APT-negative birch pollen allergic patients) before (v1) and 6–8 weeks after APT (v3) (Fig. 2, Supplemental Table E1). We found no statistically significant induction of total IgE (Fig. 2A), birch pollen-specific IgE (Fig. 2B) or rBet v 1-specific IgE levels (Fig. 2C) after APT (Supplemental Table E1). Only one subject (i.e., #10; Supplemental Table E1) who participated in the study in April shortly after the birch pollen season showed an exceptional doubling of Bet v 1-specific IgE levels in the follow up serum sample taken 8 weeks later. Subjects without birch pollen allergy (Fig. 1, group C) did not contain detectable birch pollen or rBet v 1-specific IgE (data not shown).

Since a few of the birch pollen allergic patients in our study (group 1: subjects 18, 22, 25, 26; group 2: subjects 6, 9, 10, 13) exhibited weak IgE reactivity (i.e., <20% of IgE reactivity to Bet v 1) to the mix of F1 + F2, we compared their IgE reactivity to F1, F2 and to the mix of F1 + F2 before (v1) and 6–8 weeks after APT (v3) (Supplemental Fig. 1). Only one patient (i.e., patient 10) who was tested shortly after the birch pollen season showed an increase of fragment-specific IgE binding in the follow-up serum sample. No *de novo* induction of IgE reactivity to rBet v 1 fragments after APT was noted (Supplemental Fig. 1).

### Epicutaneous application of rBet v 1 and rBet v 1 fragments induces weak increases of allergen-specific IgG antibody responses in APT-positive patients

**Figure 3:**
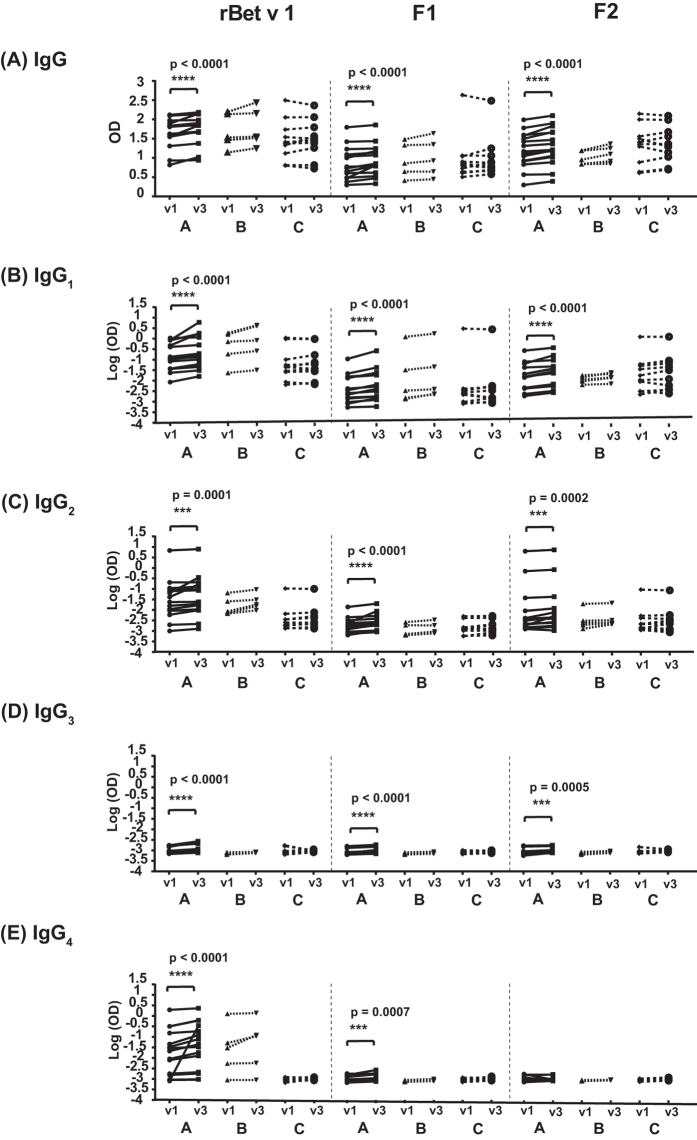
Serum IgG and IgG subclass levels to rBet v 1 and to rBet v 1 fragments (F1, F2) before and after APT. On the y-axes the optical density (OD) values for IgG **(A)** and natural log-transformed OD values for IgG_1_ **(B)** IgG_2_ **(C)** IgG_3_ **(D)** and IgG_4_ **(E)** (y-axes) to Bet v 1, F1 and F2, before (v1) and 6–8 weeks after APT application (v3) are shown. A, APT-positive birch pollen allergic patients, B, APT-negative birch pollen allergic patients and C, APT-negative subjects without birch pollen allergy (x-axes). Significant differences are indicated by asterisks (****P < 0.0001, ***P = 0.0001).

Next, we assessed the levels of IgG and the four IgG subclasses (i.e., IgG_1_, IgG_2_, IgG_3_, IgG_4_) to rBet v 1, F1 and F2 in serum samples before (v1) and 6–8 weeks after APT (v3) (Fig. 3, Supplemental Table E2). We found that epicutaneous application of rBet v 1 and rBet v 1 derivatives led to a very weak but statistically significant increase in Bet v 1- and fragment-specific IgG levels in each of the APT-positive birch pollen allergic patients (group A) (Fig. 3A, Supplemental Table E2). A similar trend without significance was observed for subjects from group B (Fig. 3A, Supplemental Table E2).

Furthermore, APT with rBet v 1 and rBet v 1 fragments also weakly but significantly boosted Bet v 1- and fragment-specific IgG and IgG subclass responses in APT-positive birch pollen allergic patients (i.e., group A) (Fig. 3B–E, respectively). Only F2-specific IgG_4_ increases did not reach statistical significance in group A. No statistically significant differences were observed for Bet v 1- or fragment-specific IgG or IgG subclass levels in sera from birch pollen allergic patients without APT reactions (i.e., group B). Likewise, subjects without birch pollen allergy and without APT reactions (i.e., group C) did not show significant increases of Bet v 1-specific IgG (Fig. 3B–E).

### Epicutaneous allergen application strongly and significantly boosts allergen-specific T cell proliferation in APT-positive patients

**Figure 4:**
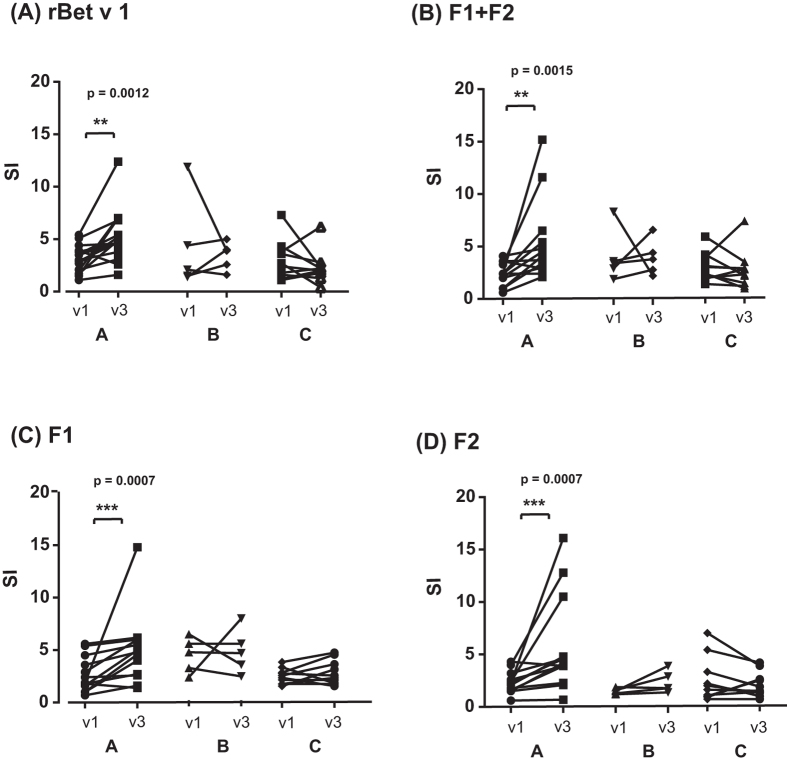
PBMC proliferation towards rBet v 1 or rBet v 1 fragments before and after APT. Shown are mean SIs (y-axes) determined from triplicate cultures of each subject in response to rBet v 1 **(A)** F1 + F2 **(B)** F1 **(C)** or F2 **(D)** before (v1) and 6–8 weeks after APT application (v3) for A, APT-positive birch pollen allergic patients, B, APT-negative birch pollen allergic patients and C, APT-negative subjects without birch pollen allergy (x-axes). Statistically significant differences are indicated (***P = 0.0001–0.001, **P = 0.001–0.01).

We then analyzed lymphocyte proliferations towards rBet v 1, F1, F2, and to the mix F1 + F2 before (v1) and 6–8 weeks after APT (v3) (Fig. 4, Supplemental Table E3). APT application strongly and significantly enhanced rBet v 1- and fragment-induced T cell proliferation in the APT-positive group A (Bet v 1: median preSI: 3.0, median postSI: 4.7; F1 + F2: median preSI: 2.4, median postSI: 4.5; F1: median preSI: 2.4, median postSI: 5.0; F2: median preSI: 2.3, median postSI: 4.2) (Fig. 4A; Supplemental Table E3). In contrast, no statistically significant change in T cell proliferation was observed in birch pollen allergic patients who were APT-negative (group B) and in APT-negative subjects without birch pollen allergy (group C) before (v1) and after (v3) APT (Fig. 4).

### Epicutaneous allergen application significantly boosts proliferation of allergen-specific skin homing T cells in APT-positive patients

**Figure 5:**
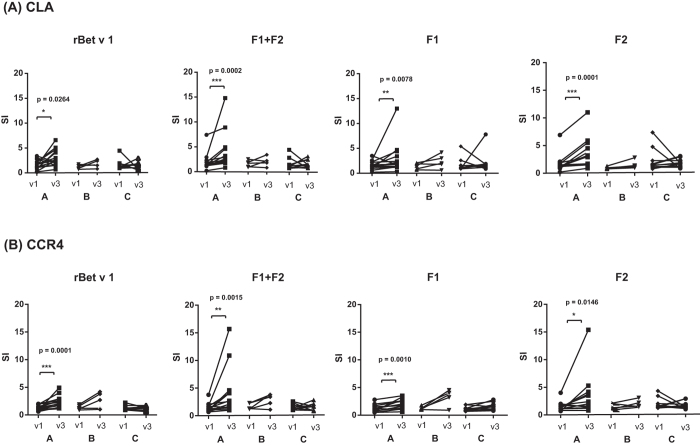
Proliferation of **(A)** CLA- and **(B)** CCR4-positive T cells towards rBet v 1 or rBet v 1 fragments before and after APT. Shown are mean SIs (y-axes) determined from triplicate cultures of each subject in response to rBet v 1, F1 + F2, F1, or F2 before (v1) and 6–8 weeks after APT application (v3) for A, APT-positive birch pollen allergic patients, B, APT-negative birch pollen allergic patients and C, APT-negative subjects without birch pollen allergy (x-axes). Statistically significant differences are indicated (***P = 0.0001–0.001, **P = 0.001–0.01, *P = 0.01–0.05).

Next, we investigated whether epicutaneous allergen application has effects on T cells expressing the skin homing markers CLA and CCR4. The proliferation of CLA- and CCR4- expressing T cells was significantly increased after APT in APT-positive patients (group A), in response to rBet v 1, F1 + F2, F1 and F2 (Fig. 5, Supplemental Tables E3–E5). For CLA, the median SIs increased from 1.4 to 2.5 for Bet v 1, from 1.4 to 2.9 for F1 + F2, from 1.4 to 1.8 for F1 and from 1.3 to 3.2 for F2 (Supplemental Table E4). For CCR4 the median SIs increased from 1.3 to 2.0 for Bet v 1, from 1.3 to 2.7 for F1 + F2, from 1.2 to 2.0 for F1 and from 1.3 to 2.0 for F2 (Supplemental Table E5). A similar trend without significance was found for APT-negative birch pollen allergic patients (group B) (Fig. 5). For APT-negative subjects without birch pollen allergy changes were heterogenous without clear trend (group C) (Fig. 5).

### Epicutaneous allergen application has distinct effects on allergen-specific cytokine responses in cultured PBMC

**Figure 6:**
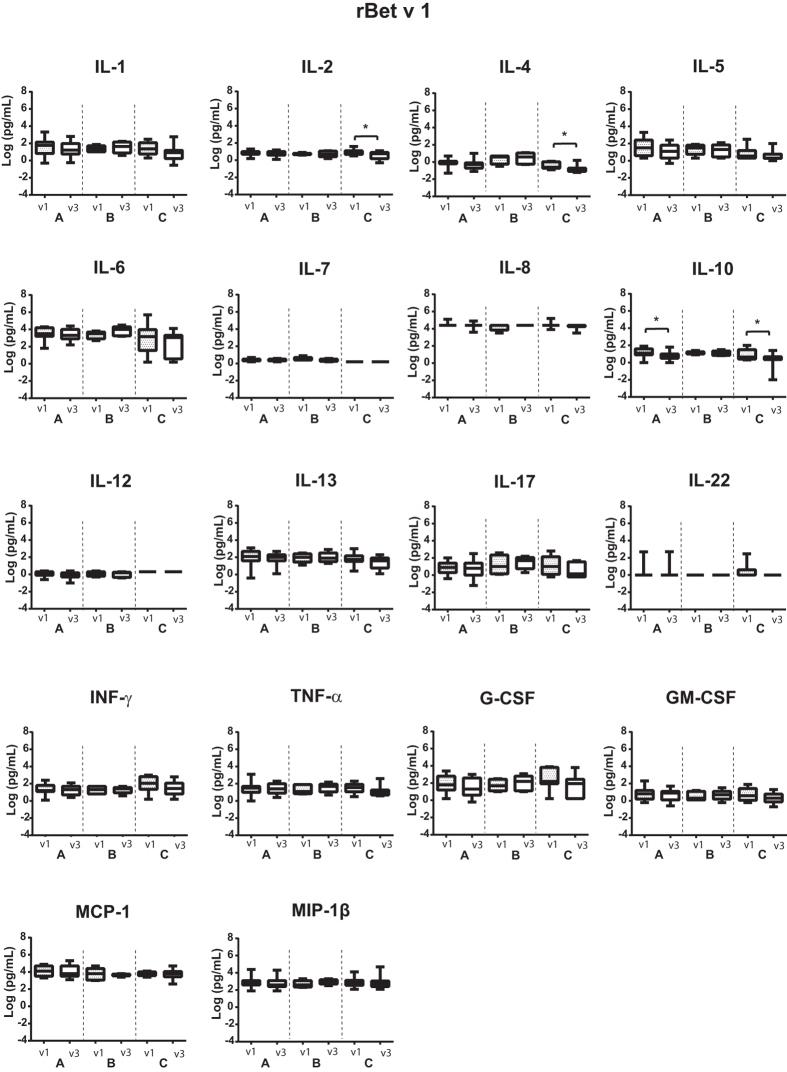
Cytokine levels measured in PBMC cultures upon stimulation with rBet v 1 before and after APT. Box plots display the mean cytokine levels as log-transformed values in pg/ml (horizontal bars: medians ± SDs) (y-axes) for subjects (**A**) APT-positive birch pollen allergic patients, (**B**) APT-negative birch pollen allergic patients and C, APT-negative subjects without birch pollen allergy before (v1) and 6–8 weeks after APT application (v3) (x-axes). Statistically significant differences are indicated (***P = 0.0001–0.001, **P = 0.001–0.01, *P = 0.01–0.05).

The effect of epicutaneous allergen application on cytokine responses was assessed by comparing the cytokine levels induced with rBet v 1, F1, F2, or the mix of F1 + F2 in PBMCs obtained from each subject before (v1) and 6–8 weeks after APT (v3) (Fig. 6, Supplemental Figs 2–4). Interestingly, after APT a significant decrease regarding the tolerogenic cytokine IL-10 was observed in supernatants of PBMCs stimulated with rBet v 1, F1, F2, and the mix of F1 + F2 in the APT-positive patients (group A) (Fig. 6, Supplemental Figs 2–4). A similar decrease in IL-10 was also observed in APT-negative subjects without birch pollen allergy (group C) but this decrease was only observed in PBMCs stimulated with rBet v 1 and the mix F1 + F2 (Fig. 6, Supplemental Fig. 4).

We also noted other statistically significant changes but these changes were heterogeneous. Upon stimulation with fragments (F1, F2, or the mix F1 + F2) but not with the folded wild-type allergen rBet v 1, a statistically significant decrease in IL-4 levels was observed in the APT-positive patients (group A) (Fig. 6, Supplemental Figs 2–4). A similar decrease was observed in patients from group C after stimulation with rBet v 1 (Fig. 6) and with rBet v 1 F1 (Supplemental Fig. 2) but not after stimulation with F2 (Supplemental Fig. 3) or F1 + F2 (Supplemental Fig. 4). Upon stimulation with F1, levels of IL-1, IL-6, IL-12, INF-γ, TNF-α, GM-CSF and MIP-1b decreased after APT in the APT-positive patients (Supplemental Fig. 2). Levels of GM-CSF significantly decreased after APT in F2- stimulated PBMC from APT-positive patients (Supplemental Fig. 3). We also noted a significant decrease of the IL-1, IL-5, IL-7, IL-12, IL-17, and INF-γ production after APT in F1 + F2-stimulated PBMCs in patients from group A (Supplemental Fig. 4). Moreover, levels of GM-CSF significantly decreased after APT in F1 + F2 stimulated PBMCs from subjects from group C (Supplemental Fig. 4).

## Discussion

Our study is the first to investigate in a longitudinal design the effects of epicutaneous application of a purified recombinant allergen and its derivatives on systemic allergen-specific antibody, T cell and cytokine responses in humans.

Despite the fact that a relatively small number of subjects were tested, statistically significant results were obtained showing that epicutaneous allergen administration by APT induced a strong and significant increase of allergen-specific T cell responses as measured by lymphocyte proliferation and by the analysis of skin homing (i.e., CCR4+, CLA+) T cells in birch pollen allergic patients who had mounted a positive APT reaction. Interestingly, Bet v 1 IgE-positive subjects as a group generally had larger increases of T cell proliferation at v3 whereas only certain Bet v 1 IgE-negative subjects showed increases and there was no increase regarding the median proliferation of the latter group. However, no association of increases in Bet v 1-specific T cell proliferation with the levels of Bet v 1-specific IgE was found. We also looked into patients with high increases of Bet v 1-specific T cell proliferation (e.g., subjects 1, 21, 19, 9, 13 Supplemental Table 3) but did not find evidence that these subjects had stronger APT reactions to Bet v 1 than the other patients of group A or higher total or Bet v 1-specific IgE levels. Notably, high lymphocyte proliferation levels were observed at visit 1 for some subjects without IgE sensitization to birch pollen (Supplementary Table E3, group C). This is in accordance with several other studies reporting that subjects without IgE sensitization to a given allergen can exhibit allergen-specific T cell responses^10–13^.

Accordingly we found that subjects without IgE-sensitization to birch pollen (group C) mounted Bet v 1-specific IgG responses as a “natural” IgG response, which requires T cell help. This is quite common and has been described many times and recently in a large population study^14^.

**Figure 7:**
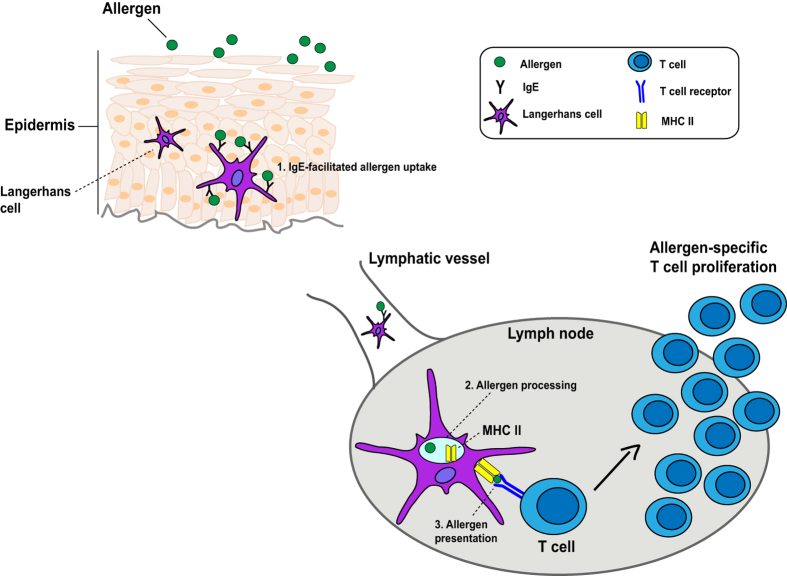
Hypothetical scheme for the boosting of systemic allergen-specific T cell responses by epicutaneous allergen application.

Another observation was that epicutaneous allergen administration had no relevant effects on allergen-specific IgG or IgE antibody production. Only in one patient, who was tested shortly after the birch pollen season, an increase of Bet v 1-specific IgE was noted which most likely was caused by a boost through seasonal natural allergen exposure. The increase of allergen-specific IgG levels was statistically significant again only in the APT-positive birch pollen allergic patients. Although statistically significant, the increase of allergen-specific IgG levels in group A was very weak because it accounted only for less than 2% (median OD increase of 0.04) of that observed for a positive control serum from a patient who had received subcutaneous AIT (OD increase of 0.9). We therefore think that the increase of IgG was not relevant. This finding may have important implications for epicutaneous allergen-specific immunotherapy because the induction of allergen-specific IgG is considered as a major mechanism of successful AIT. In another recent study, Cabauatan *et al*., observed in a guinea pig model that even repetitive epicutaneous patch vaccination with rBet v 1 without adjuvant did not induce relevant rBet v 1-specific IgG levels in guinea pigs^15^. Allergen-specific IgG responses were only induced when heat-labile enterotoxin was used as adjuvant^15^. Based on the results obtained in our study one might propose the mechanism depicted in Fig. 7. Since increases of T cell responses occurred mainly in patients with APT reactions containing Bet v 1-specific IgE one might assume that intact Bet v 1 was picked up by antigen presenting cells (e.g., Langerhans cells (LCs) and/or dendritic cells) containing Bet v 1-specific IgE by IgE-facilitated allergen presentation in the epidermis, most likely via FcɛRI. The Bet v 1 fragments which were also applied by APT showed almost no IgE reactivity and therefore may play a minor or no role in the boosting of the T cell response. Then Bet v 1-loaded LCs may migrate to regional lymph nodes where they boost allergen-specific T cells, among them T cells homing to the skin. A possible explanation for the selective induction of allergen-specific T cell responses and the lack (IgE) or weak (IgG) induction of antibody responses might be that most of the allergen has been processed by the LCs and appears only as T cell-reactive peptide in the context of MHC class II on their surface, whereas not much intact allergen is left which could interact with Bet v 1-specific B cells expressing surface IgE or IgG recognizing only intact allergen. In fact, a similar observation was made earlier when dendritic cells were pulsed with allergen and then used to sensitize rats. Also in this model only the development of allergen-specific T cell responses were observed but no allergen-specific antibody production^16^. It is also possible that Bet v 1-sensitized allergic patients had a larger pool of Bet v 1-specific T cells which may be activated also by simple phagocytosis. However, there was no significant difference regarding Bet v 1-specific T cell proliferation between allergic patients with (i.e., groups 1, 2) and without Bet v 1-specific IgE (i.e., group 3) at the time of APT administration which speaks against this hypothesis^6^. The allergen-specific immune response boosted by epicutaneous allergen administration is different from that induced by other routes, although there are some similarities for example with sublingual or oral vestibule administration involving the oral mucosa. In fact, in an elegant study, Allam *et al*., observed that the oral mucosa is rich in LCs expressing FcεRI^17^ and they showed that allergen contact of LCs enhances their migratory properties^18^. However, unlike epicutaneous administration, sublingual and oral vestibule allergen contact strongly increased (almost 3-fold) allergen-specific IgE levels^19^. The induction of allergen-specific IgG after oral allergen contact was as low as observed by us for the epicutaneous route. Allergen contact with the oral mucosa when applied in the form of sublingual and vestibular administration thus resembles more the features of nasal allergen exposure. In fact, we had found earlier that a single nasal allergen application strongly boosted systemic allergen-specific IgE responses and had only negligible effects on allergen-specific IgG production^7, 8, 20^. The boost of systemic allergen-specific IgE production by nasal application required intact folded allergen which can bind to IgE antibodies and was not obtained by nasal application of unfolded allergen fragments which lacked IgE reactivity^8^. In this context it will be interesting to investigate in EPIT studies if more frequent and prolonged epicutaneous allergen exposure may have stronger effects on the induction of allergen-specific antibody responses.

The boost of allergen-specific T cell responses was observed for skin-homing T cells and it is thus quite possible that these cells may be also important for the induction of skin inflammation in the patients. In fact, the analysis of allergen-induced cytokine responses showed that patients with APT reactions produced significantly less of the tolerogenic cytokine, IL-10 after APT when their PBMC were stimulated with Bet v 1 and the Bet v 1 fragments but this was also observed in the group not allergic to birch.

Data from studies conducted in murine models have suggested highly divergent effects of epicutaneous administration of allergens. Some mouse model studies suggest that epicutaneous allergen administration increases allergic inflammation^21–24^. By contrast, other studies indicate that it has positive effects on allergic responses^25–28^. We show in a clinical study that epicutaneous administration of allergen to allergic subjects boosts allergen-specific systemic T cell responses. Our study thus indicates that data from animal models cannot always be transferred to the human situation and that it is very important to analyse the effects of the administration of allergens via different routes on immune responses in pilot clinical trials in allergic patients. Such pilot trials will be important to explore new routes and adjuvants for AIT.

## Methods

### Study design and ethics statement

This was a registered monocentric study conducted at the Department of Dermatology of the Medical University of Vienna (ClinicalTrials.gov number: NCT02098551, date of registration: July 26, 2011; EudraCT number: 2009-011859-51), approved by the Ethics Committee of the Medical University of Vienna (EK147/2009) and by the Austrian Health authorities. All experiments were performed in accordance with relevant guidelines and regulations of the Declaration of Helsinki. Participants signed informed consent before inclusion in the study. In total 30 adult subjects (16 female and 14 male) between the ages of 18 and 65 years were enrolled (Fig. 1). Fifteen suffered from birch pollen-related atopic dermatitis (AD) (8 female and 7 male, mean age 28.7 ± 6.4 years) (Group 1), 5 had birch pollen-related rhinoconjunctivitis without history of AD (2 female and 3 male, mean age 34.4. ± 9.3 years) (Group 2), 5 were allergic patients without birch pollen allergy (2 female and 3 male, mean age 30.8. ± 1.9 years) (Group 3), and 5 were non-allergic individuals (4 female and 1 male, mean age 29.8. ± 5 years) (Group 4) (Fig. 1)^6^.

At visit 1, blood samples were obtained before ATP for baseline detection of IgE and IgG antibodies as well as for investigation of cellular (i.e., T cell proliferation, CLA+ and CCR4+ T cell proliferation) and cytokine responses. Immediately thereafter, subjects underwent atopy patch testing (APT) (Fig. 1). APT was performed according to the standardized protocol for APT established by the European Task Force on Atopic Dermatitis (ETFAD)^1^ on clinically uninvolved skin on the back of each of the subjects with 160 μg of rBet v 1, 160 μg of rBet v 1 F1, 160 μg of rBet v 1 F2 and with an equimolar mix containing 80 μg of each rBet v 1 fragment^6^. Purified folded recombinant Bet v 1 (batch#Bet v 1a-GMP-0802), hypoallergenic rBet v 1 fragment 1 (F1: amino acids 1–74) (batch#Bet v 1a F1-GMP-0803) and hypoallergenic rBet v 1 fragment 2 (F2: amino acids 75–160) (batch#Bet v 1a F2-GMP-0801) produced according to GMP guidelines were purchased from Biomay AG (Vienna, Austria).

The skin of the subjects was stripped with a tape and the substances were applied for 48 h using aluminium cups (12 mm diameter; Finn Chambers on Scanpor, Large, Epitest Ltd Oy). Pure Vaseline petroleum jelly (Unilever, London, United Kingdom) served as negative control. After 48 hours (day 3, visit 2) APTs were read and photo-documented. Grading of positive APT reactions was done according to the European Task Force on Atopic Dermatitis (ETFAD)^1^.

In this follow-up study we called in the study subjects six to eight weeks after APT and compared humoral, cellular immune responses and cytokine responses at visit 1 and visit 3 in subjects who were APT-positive birch pollen allergic patients (i.e., group A) (rBet v 1 positive APT: n = 15; F1 + F2 positive APT: n = 13), APT-negative birch pollen allergic patients (i.e., group B) (n = 5) and in APT-negative subjects without birch pollen allergy (i.e., group C) (n = 10) (Fig. 1).

The systemic immune responses at baseline (visit 1) were compared with those at a third visit (6–8 weeks after APT). The third visit for the assessment of systemic immune responses was planned 6–8 weeks after APT because we have observed that nasal allergen administration led to a robust increase of systemic antibody production at this time point^20^.

### Determination of IgE antibodies and IgE reactivity

Total IgE and allergen-specific IgE to birch pollen extract and to rBet v 1 were determined in serum samples obtained at visit 1 shortly before APT and 6–8 weeks after APT (visit 3) using quantitative ImmunoCAP (Phadia, Uppsala, Sweden) measurements. IgE reactivity to rBet v 1 fragment 1 (F1), rBet v 1 fragment 2 (F2) and to the equimolar mix of the rBet v 1 fragments (F1 + F2) was analysed in a RAST-based non-denaturing dot blot assay^29^.

### Measurement of specific IgG and IgG subclass reactivity

Allergen-specific IgG and IgG subclass responses were measured by ELISA (see supplementary material #1) and IgG subclass were analyzed as log-transformed values.

### Proliferation of PBMC, CLA^+^ and CCR4^+^ T cells as well as *in vitro* cytokine production

Heparinized blood samples were obtained from each of the study subjects at visit 1 and visit 3. PBMCs were isolated by centrifugation over a Ficoll-Paque PLUS gradient (Amersham, GE Healthcare, Buckinghamshire, UK) and cultured (2 × 10^6^) in triplicates in 96-well round-bottom tissue-culture plates (Thermo Fischer Scientific, Roskilde, Denmark) in 200 µL Ultra Culture Medium (Lonza, Verviers, Belgium) supplemented with 2mM L-Glutamine, 50 µM β-mercaptoethanol and 0.1 mg/mL gentamicin (GIBCO, Invitrogen, USA) in the presence of either rBet v 1, rBet v 1 F1, rBet v 1 F2, or rBet v 1 fragments in an equimolar mix (F1 + F2) (5 µg/well) (Biomay AG, Vienna, Austria). IL-2 (4 U/well) (Roche diagnostics GmbH, Mannheim, Germany) served as positive control and medium alone as negative control (six replicate wells each). After 6 days of incubation at 37 °C, cells were pulsed with 0.5 μCi/well [^3^H] thymidine (Perkin Elmer, Boston, MA) for 16 hours. The incorporation of radioactivity and the calculation of the results as stimulation index were performed as described^30^. Allergen-specific responses of CCR4^+^ and CLA^+^ cells were analyzed by carboxyfluorescein diacetate succinimidyl (CSFE) staining (see supplementary material #2). The levels of cytokines IL-1, IL-2, IL-4, IL-5, IL-6, IL-7, L-8, IL-10, IL-12, IL-13, IL-17, INF-γ, TNF-α, C-GSF, GM-CSF, MCP-1, and MIP-1b were measured in supernatants of PBMCs cultures which were stimulated identically as for the proliferation assays at visit 1 and visit 3 using a Bio-Plex Pro^TM^ human cytokine 17-plex immunoassay (Bio-Rad Inc., Hercules, CA). A Human IL-22 FlowCytomix Simplex kit (eBiosciences, San Diego, CA) was used for the measurement of IL-22 levels. Due to their skewed distributions log-transformed values of cytokine levels were used for statistical analyses. Results are displayed as log-transformed concentrations (pg/mL).

### Statistics analysis

Differences between specific immune responses measured at visit 1 and visit 3 after APT were evaluated using the non-parametric Wilcoxon signed-rank test. Skewed data (i.e., cytokine levels and IgG subclass levels) were log-transformed. A *P* value < 0.05 was considered as statistically significant.

## Electronic supplementary material

Supplementary Information
