# Supplementary material for: Epicutaneous allergen application preferentially boosts specific T cell responses in sensitized patients
Source: Sci Rep. 2017 Sep 14;7:11657. doi: 10.1038/s41598-017-10278-1 (PMC5599525; doi:10.1038/s41598-017-10278-1)

Epicutaneous allergen application preferentially boosts specific T cell responses in sensitized patients

Raffaela Campana[1], Katharina Moritz[2], Angela Neubauer[3], Hans Huber[3], Rainer Henning[3], Tess M. Brodie[4], Alexandra Kaider[5], Federica Sallusto[4,6], Stefan Wöhrl[2] & Rudolf Valenta[1]*

[1] Division of Immunopathology, Department of Pathophysiology and Allergy Research, Center of Pathophysiology, Infectiology and Immunology, Vienna General Hospital (AKH), Medical University of Vienna, Austria; [2] Division of Immunology, Allergy and Infectious Diseases (DIAID), Department of Dermatology, Vienna General Hospital (AKH), Medical University of Vienna, Austria; [3] Biomay AG, Vienna Competence Center, Austria; [4] Cellular Immunology Laboratory, Institute for Research in Biomedicine, Bellinzona, Switzerland; [5] Center for Medical Statistics, Informatics and Intelligent Systems, Section for Clinical Biometrics, Medical University of Vienna, Austria; [6] Institute of Microbiology, ETH Zuerich, Switzerland

Supplemental Materials

Supplemental Material #1:

Measurement of specific IgG and IgG subclass reactivity

ELISA plates (Greiner, Kremsmünster, Austria) were coated with purified rBet v 1, F1 or F2 dissolved in carbonate buffer, pH 9.6, at a concentration of 5µg/mL by incubation at 4°C overnight. Plates were washed three times with PBS-T (PBS + 0.05 % Tween 20) and blocked with 2 % bovine serum albumin (BSA) (Roth, Karlsruhe, Germany) in PBS-T at 4°C for 6 hours. ELISA plate-bound allergens (triplicate wells) were exposed to each patients' serum diluted in PBS-T + 0.5 % BSA (1:50 for IgG and 1:25 for IgG_{1-4} detection) at 4°C overnight. After washing five times with PBS-T, bound human IgGs were detected with rabbit anti-human IgG antibodies (Jackson ImmunoResearch Laboratories, West Grove, PA) or with mouse monoclonal anti-human IgG_{1-4} antibodies[1] diluted 1:4000 and 1:1000 in PBS-T + 0.5 % BSA, respectively. After overnight incubation at 4°C, plates were again washed 5 times with PBS-T and bound human IgG antibodies were detected with an anti-rabbit IgG Horseradish Peroxidase linked whole antibodies from donkey (for total IgG) and anti-mouse IgG Horseradish Peroxidase-linked whole antibodies from sheep (for IgG subclasses) (GE Healthcare, UK Limited) for 1 hour at 37°C and 4°C. Plates were washed four times with PBS-T and the color reaction was determined by addition of staining solution ABTS (2,2'-Azino-bis(3-ethylbenzthiazoline-6-sulfonic acid) diammonium salt; Sigma-Aldrich, St.Louis, Missouri, USA) (100µl/well). The optical density was measured using an ELISA Reader (Dynatech, Denkendorf, Germany) at 405 nm. Results are displayed as mean OD values of triplicate determinations with an SD of less than 5% for IgG for the IgG subclass (log-transformed) responses. Plate-to-plate normalization was obtained by including a positive serum control (serum from a patient who had undergone birch pollen AIT) and a negative control (buffer without serum) on each of the ELISA plates.

Supplemental Material #2:

Proliferation of PBMC, CLA^{+} and CCR4^{+} T cells as well as *in vitro* cytokine production

PBMCs obtained at visits 1 and 3 were also labelled with carboxyfluorescein diacetate succinimidyl (CSFE) (Invitrogen, Oslo, Norway) for 10 minutes at 37°C in a concentration of 1mL of 5μM CFSE solution per 10 x 10^{6} cells. CFSE-labeled PBMCS (2 x 10^{6}) were then cultured in 96-well round-bottom tissue-culture plates (Thermo Fischer Scientific, Roskilde, Denmark) together with rBet v 1, rBet v 1 F1, rBet v 1 F2 or rBet v 1 fragments mix for 7 days as described for the proliferation assay. Medium alone (Lonza, Verviers, Belgium) and 3μL/well of Dynabeads containing anti-CD3 and anti-CD28 (Invitrogen, Carlsbad, CA) were used as negative and positive controls, respectively. Experiments were performed in triplicate wells. Cells were centrifuged and stained (20 minutes on ice) in a total of 50 μL fluorescence-activated cell sorting (FACS) buffer (PBS, 0.01 % wt/vol NaN_3, 1% wt/vol BSA) containing 7-amino-actino-mycin D (7-AAD) (3μL/well) (Biolegend, San Diego, CA) plus either biotin-labelled anti-CCR4 or anti-CLA antibodies (BD Bioscience, San Jose, CA) (10 μL/well). A rat IgM isotype (CLA) and a mouse IgG_1 isotype (CCR4) (BD Bioscience, San Jose, CA) were used as negative controls. Finally, cells were stained as above with 1 μL/well Streptavidin-Phycoerythrin-Cyanine 7 (PC7-Streptavidin) (BD Bioscience, San Jose, CA), and then proliferation of CLA+ and CCR4+ T lymphocytes was assessed by flow cytometry using a Cytomics FC 500 flow cytometer (Beckman Coulter, Fullerton, CA). Data were analyzed using FlowJo 7.2.5 software. Cells evaluated for CLA and CCR4 were gated according to FSC/SSC profile and dead cells were excluded by 7-AAD viability staining (Biolegend). Results were expressed as the mean percentages of CLA+CD3+ and CCR4+CD3+ cells and displayed as stimulation index (SI). The stimulation index was calculated as the quotient of the mean percentages of CD3+ cells expressing CLA or CCR4 in stimulated and in unstimulated cultures.

References

1. Stern, D.A. *et al*. Exposure to a farming environment has allergen-specific protective effects on TH2-dependent isotype switching in response to common inhalants. *J Allergy Clin Immunol* **119**, 351-358 (2007).

Supplemental Figures and Tables

SUPPLEMENTAL FIG 1. IgE reactivities to rBet v 1 fragments before and after APT. Sera obtained from the study subjects (n=30) before (v1) and 6-8 weeks after APT application (v3) were tested for IgE-reactivity to dot-blotted F1, F2, a mix of F1+F2 and BSA. Buffer (BC) served as negative control. Bound IgE antibodies were detected with ^{125}I-labeled anti-human IgE Abs and visualized by autoradiography.

SUPPLEMENTAL FIGS 2-4. Cytokine levels measured in PBMC cultures upon stimulation with F1 (Fig 2), F2 (Fig 3) or F1+F2 (Fig 4) before and after APT. Box plots display the log-transformed mean cytokine levels in pg/ml (horizontal bars: medians ± SDs) (y-axes) for subjects A, APT-positive birch pollen allergic patients, B, APT-negative birch pollen allergic patients and C, APT-negative subjects without birch pollen allergy before (v1) and 6-8 weeks after APT application (v3) (x-axes). Statistically significant differences are indicated (***P = 0.0001-0.001, **P = 0.001-0.01, *P = 0.01-0.05).

SUPPLEMENTAL TABLE 1. Total IgE, birch pollen-specific IgE, rBet v 1-specific IgE measured in sera from group A, APT-positive birch pollen allergic patients and group B, APT-negative birch pollen allergic patients before (v1) and 6-8 weeks after APT application (v3).

SUPPLEMENTAL TABLE 2. IgG levels specific for rBet v 1, F1 and F2 measured in sera from group A (APT-positive birch pollen allergic patients), group B (APT-negative birch pollen allergic patients) and group C (APT-negative subjects without birch pollen allergy) before (v1) and 6-8 weeks after APT application (v3).

SUPPLEMENTAL TABLES 3-5. Proliferative responses (SIs) of blood PBMC (Table 3), CLA-positive T cells (Table 4) and CCR4-positive T cells (Table 5) to rBet v 1, F1, F2 and F1+F2 from group A (APT-positive birch pollen allergic patients), group B (APT-negative birch pollen allergic patients) and group C (APT-negative subjects without birch pollen allergy) before (v1) and 6-8 weeks after APT application (v3).

	Total IgE (kU/l)		Birch-specific IgE (kUA/l)		Bet v 1-specific IgE (kUA/l)	
**Group A**	**Visit 1**	**Visit 3**	**Visit 1**	**Visit 3**	**Visit 1**	**Visit 3**
1	934	754	3.52	3.7	5.5	4.48
5	55.6	50.6	0.72	0.8	0.76	0.84
7	84.2	76.6	8	7.44	5.76	5.1
8	590	490	11.38	8.82	12.82	12
15	522	562	37.4	37.6	41.2	42
18	4788	4702	100.2	80.6	93.4	76.6
20	240	228	31.2	24.8	6.62	5.3
21	248	240	7.1	3.2	6.78	3.02
22	742	670	46.8	32.2	49	37.2
26	3198	3296	122.4	133	125.6	136
19	446	560	8.26	9.7	7	5.6
9	90.8	83.2	6.6	5.78	6.44	5.96
10	44.6	50.6	4.76	9.52	3.18	6.32
13	392	386	47.4	55.8	61	56.8
11	42	50.2	12	15.1	13.78	17.38
Median	392	386	11.38	9.7	7	6.32
**Group B**						
24	13840	14098	52.8	63.8	30.2	31.8
25	29	44.4	13.24	16.4	13.6	16.24
27	388	320	36.4	25.4	39	27
28	17.96	21.6	0.7	0.7	0.7	0.8
6	412	328	36	29	39.4	31.2
Median	388	320	36	25.4	30.2	27

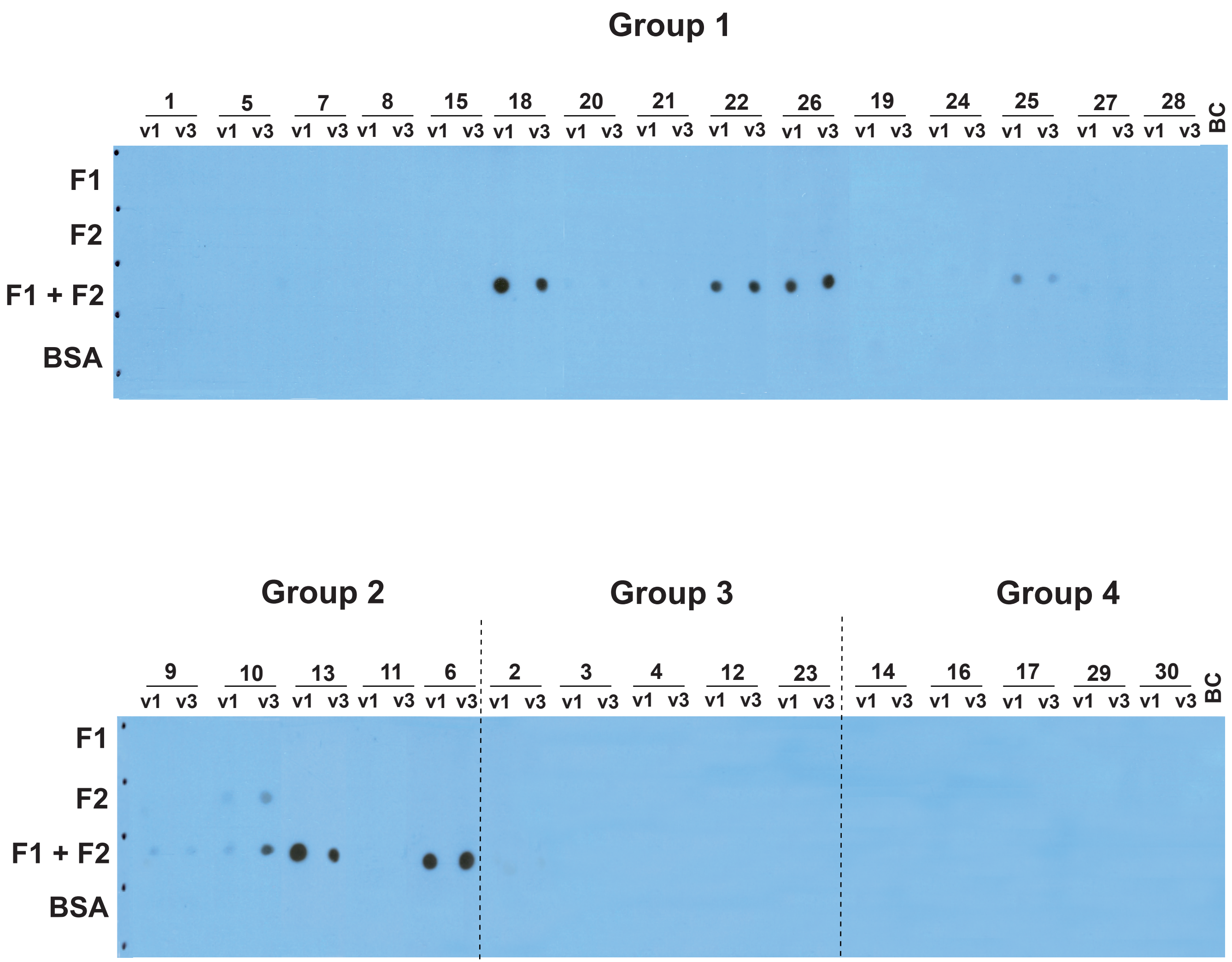
Group 1
1
5
7
8
15
18
20
21
22
26
19
24
25
27
28
v1 v3
BC
F1
F2
F1 + F2
BSA
Group 2
Group 3
Group 4
9
10
13
11
6
2
3
4
12
23
14
16
17
29
30
v1 v3
BC
F1
F2
F1 + F2
BSA

Supplemental Table E2

	IgG levels (OD)					
	rBet v 1		F1		F2	
Group A	Visit 1	Visit 3	Visit 1	Visit 3	Visit 1	Visit 3
1	1.550	1.685	0.769	0.785	1.511	1.683
5	0.933	0.942	0.602	0.613	0.580	0.589
7	0.820	1.021	0.373	0.468	0.311	0.407
8	1.309	1.375	0.294	0.326	0.928	1.037
15	1.624	1.685	1.030	1.070	1.439	1.482
18	1.805	1.858	1.416	1.431	1.182	1.221
20	1.600	1.663	0.470	0.801	1.326	1.380
21	1.825	1.844	0.646	0.791	1.792	1.911
22	1.893	2.149	0.996	1.173	1.590	1.842
26	1.974	1.981	1.223	1.240	0.942	1.065
19	1.550	1.842	0.481	0.525	1.062	1.206
9	1.818	1.865	0.728	0.846	0.843	0.902
10	2.095	2.138	1.792	1.852	2.004	2.112
13	1.836	1.907	0.753	0.811	1.127	1.191
11	2.118	2.183	0.853	0.924	0.967	1.110
Median	1.805	1.844	0.753	0.811	1.127	1.206
Group B						
24	1.531	1.553	1.476	1.634	0.856	0.928
25	2.134	2.167	1.326	1.344	1.200	1.264
27	1.463	1.519	0.641	0.642	0.852	0.863
28	1.142	1.254	0.853	0.924	0.967	1.110
6	2.194	2.443	0.406	0.443	1.207	1.378
Median	1.531	1.553	0.853	0.924	0.967	1.110
Group C						
2	1.331	1.564	0.725	0.888	1.572	1.686
3	1.376	1.392	1.056	1.054	1.368	1.377
4	0.801	0.727	0.603	0.689	0.624	0.710
12	1.119	1.262	1.036	1.244	0.898	1.042
23	1.410	1.457	0.639	0.659	1.422	1.568
14	2.053	2.060	0.898	0.870	2.162	2.108
16	1.541	1.501	0.795	0.745	1.311	1.156
17	1.736	1.796	0.500	0.573	2.011	2.001
29	0.812	0.813	0.844	0.772	0.650	0.732
30	2.496	2.365	2.628	2.481	1.511	1.305
Median	1.393	1.479	0.819	0.821	1.395	1.341

Supplemental Table E3

	PBMC Proliferation (SIs)							
	rBet v 1		F1+F2		F1		F2	
**Group A**	**Visit 1**	**Visit 3**	**Visit 1**	**Visit 3**	**Visit 1**	**Visit 3**	**Visit 1**	**Visit 3**
1	1.7	4.9	3.3	11.6	1.0	4.6	4.0	12.8
5	3.9	2.6	3.6	2.9	1.8	2.7	4.3	3.9
7	3.0	3.8	1.0	3.4	4.5	5.5	1.5	2.1
8	3.5	4.7	1.0	4.1	0.7	1.6	0.6	0.7
15	2.1	3.1	2.0	3.0	2.4	2.6	2.6	3.9
18	5.1	6.8	4.1	4.8	5.4	6.0	2.3	4.6
20	3.8	4.7	3.6	3.7	3.6	4.8	2.1	4.8
21	2.7	5.2	2.4	6.5	2.9	6.0	1.6	3.9
22	3.6	5.4	2.1	4.5	5.6	6.2	1.5	4.2
26	2.9	4.7	2.4	2.4	1.0	4.0	3.2	4.6
19	1.5	7.0	3.8	5.1	3.1	6.0	2.8	3.6
9	2.0	7.1	2.4	5.4	1.6	5.0	2.4	10.5
10	1.1	1.6	0.6	2.1	1.8	1.4	1.8	2.3
13	5.4	12.4	2.4	15.2	1.9	14.8	2.3	16.1
11	4.4	4.6	4.3	5.5	2.9	5.6	3.4	6.2
Median	3.0	4.7	2.4	4.5	2.4	5.0	2.3	4.2
**Group B**								
24	1.5	2.6	2.9	6.6	2.2	7.8	1.3	1.8
25	1.4	4.0	3.4	3.8	4.6	4.5	1.9	1.8
27	2.1	1.6	1.9	2.8	3.1	2.3	1.2	1.4
28	4.4	5.0	3.6	4.4	5.4	5.4	1.5	3.9
6	11.9	3.9	8.3	2.2	6.3	3.4	1.6	2.9
Median	2.1	3.9	3.4	3.8	4.6	4.5	1.5	1.8
**Group C**								
2	3.6	2.8	2.9	2.8	1.7	1.7	0.8	2.5
3	3.8	6.2	3.8	7.3	3.8	4.7	6.9	3.8
4	4.3	2.2	3.0	2.7	3.3	1.8	5.3	4.1
12	7.3	2.3	5.8	3.4	2.8	4.5	3.2	1.8
23	2.4	2.0	2.2	1.4	2.1	2.1	1.9	1.2
14	1.1	1.8	4.1	2.1	2.7	2.5	1.0	1.3
16	1.2	2.0	1.9	2.2	1.5	3.1	0.6	0.6
17	2.7	0.4	2.3	0.9	2.3	1.5	2.1	0.9
29	1.8	2.2	1.8	2.7	2.5	3.6	0.9	2.4
30	1.6	1.3	1.3	1.1	1.7	2.3	1.5	1.4
Median	2.6	2.1	2.6	2.5	2.4	2.4	1.7	1.6

Supplemental Table E4

	Proliferation of CLA+ T cells (SIs)							
	rBet v 1		F1+F2		F1		F2	
Group A	Visit 1	Visit 3	Visit 1	Visit 3	Visit 1	Visit 3	Visit 1	Visit 3
1	0.3	2.5	1.1	2.6	1.4	1.4	0.9	3.8
5	1.2	2.3	1.4	2.1	1.3	1.4	1.2	1.5
7	1.1	1.5	1.4	1.9	1.0	1.2	1.4	1.6
8	0.2	0.7	0.2	0.8	0.2	0.3	0.2	0.9
15	2.2	4.7	7.4	8.9	1.0	4.7	6.9	11.0
18	2.2	5.1	2.3	2.9	2.6	13.0	1.7	5.5
20	1.4	6.6	1.3	3.2	1.5	1.8	1.0	4.5
21	1.8	3.0	1.9	4.9	1.7	1.8	1.1	3.2
22	2.8	1.5	1.9	2.3	3.5	1.7	1.2	3.0
26	1.7	2.6	1.4	2.3	1.6	3.4	1.5	1.6
19	0.8	4.3	1.2	5.2	1.3	4.3	1.3	4.5
9	1.3	1.5	1.2	1.7	0.6	1.3	1.5	1.7
10	0.9	4.1	1.3	4.6	1.2	2.2	1.3	3.2
13	3.4	2.2	2.9	14.8	2.2	2.4	2.1	5.9
11	3.0	2.0	1.8	7.8	2.3	4.4	1.9	2.9
Median	1.4	2.5	1.4	2.9	1.4	1.8	1.3	3.2
Group B								
24	1.3	1.5	2.5	2.1	1.5	4.2	0.9	1.2
25	1.2	2.6	1.9	3.4	2.1	3.0	1.2	2.7
27	0.8	2.3	0.9	2.2	0.9	2.0	0.8	1.3
28	1.6	1.4	1.7	1.8	1.9	1.8	0.7	0.9
6	0.6	0.7	1.0	0.8	0.7	0.6	0.9	1.1
Median	1.2	1.5	1.7	2.1	1.5	2.0	0.9	1.2
Group C								
2	0.9	1.9	0.8	1.5	1.2	1.5	0.6	1.5
3	1.0	1.7	1.5	2.6	1.5	1.7	1.5	2.9
4	1.2	0.9	1.0	0.8	0.9	7.8	1.3	1.5
12	1.1	2.9	1.5	2.5	1.1	1.9	1.4	2.8
23	1.3	1.8	1.2	1.6	1.2	1.5	1.2	1.7
14	1.2	1.6	1.1	3.2	1.0	1.7	2.1	2.1
16	4.4	0.7	4.5	1.5	5.4	1.8	7.2	1.6
17	1.9	0.5	2.9	0.9	2.6	0.9	4.6	0.7
29	1.7	1.4	1.3	1.3	1.6	1.2	1.0	1.1
30	0.9	1.0	0.8	1.0	0.9	1.2	0.9	0.9
Median	1.2	1.5	1.3	1.5	1.2	1.6	1.3	1.6

Supplemental Table E5

	Proliferation of CCR4+ T cells (SIs)							
	rBet v 1		F1+F2		F1		F2	
Group A	Visit 1	Visit 3	Visit 1	Visit 3	Visit 1	Visit 3	Visit 1	Visit 3
1	0.9	1.1	1.1	1.1	0.5	1.1	1.1	1.2
5	1.0	1.2	0.8	1.7	0.8	1.3	1.2	1.2
7	1.0	1.7	0.9	2.1	0.7	1.6	1.1	2.0
8	0.6	1.4	0.7	1.0	0.5	0.6	0.8	0.8
15	1.9	4.0	3.8	15.7	2.0	2.6	4.0	15.4
18	1.5	2.5	2.1	2.6	2.8	3.5	1.5	3.5
20	1.1	2.0	1.0	4.1	0.7	3.3	0.7	5.3
21	2.0	2.2	1.7	4.1	1.5	1.7	1.3	3.8
22	1.4	1.9	1.7	1.5	1.2	2.0	2.0	1.4
26	1.9	2.9	1.4	2.7	1.7	2.5	1.2	1.3
19	2.1	2.6	3.3	3.7	1.9	3.8	2.0	2.7
9	1.1	1.1	1.3	1.4	1.2	1.0	1.8	2.4
10	0.8	4.9	1.0	4.6	1.0	2.7	1.3	4.2
13	1.3	2.0	1.8	10.9	1.5	1.8	1.7	1.6
11	1.4	2.5	1.3	4.6	1.0	3.3	1.7	1.9
Median	1.3	2.0	1.3	2.7	1.2	2.0	1.3	2.0
Group B								
24	1.2	1.2	1.2	3.9	1.1	3.7	0.9	1.4
25	1.9	4.2	2.3	3.8	1.8	4.0	2.1	3.1
27	1.1	2.7	2.3	3.4	1.2	4.5	1.0	2.2
28	1.6	3.8	1.2	2.3	1.3	3.2	1.3	1.6
6	0.9	1.0	1.3	1.1	1.0	0.9	2.1	1.5
Median	1.2	2.7	1.3	3.4	1.2	3.7	1.3	1.6
Group C								
2	0.9	0.9	0.9	0.9	0.9	0.9	1.2	1.2
3	1.0	1.7	0.9	2.0	0.8	1.9	1.2	2.8
4	0.9	0.8	0.9	0.8	0.7	0.8	1.4	1.0
12	1.0	1.3	1.2	2.7	1.1	1.6	1.4	2.8
23	1.0	1.2	1.1	1.5	0.9	1.5	1.1	1.6
14	0.9	0.6	2.2	0.7	0.8	1.1	3.3	0.9
16	2.2	1.3	2.4	1.5	1.9	2.5	4.2	1.3
17	1.2	1.5	1.4	1.6	1.2	2.8	1.9	1.8
29	1.7	1.7	1.7	1.5	1.9	1.5	1.1	1.4
30	1.3	1.2	1.2	0.9	1.3	1.1	1.6	1.1
Median	1.0	1.3	1.2	1.5	1.0	1.5	1.4	1.4

F1

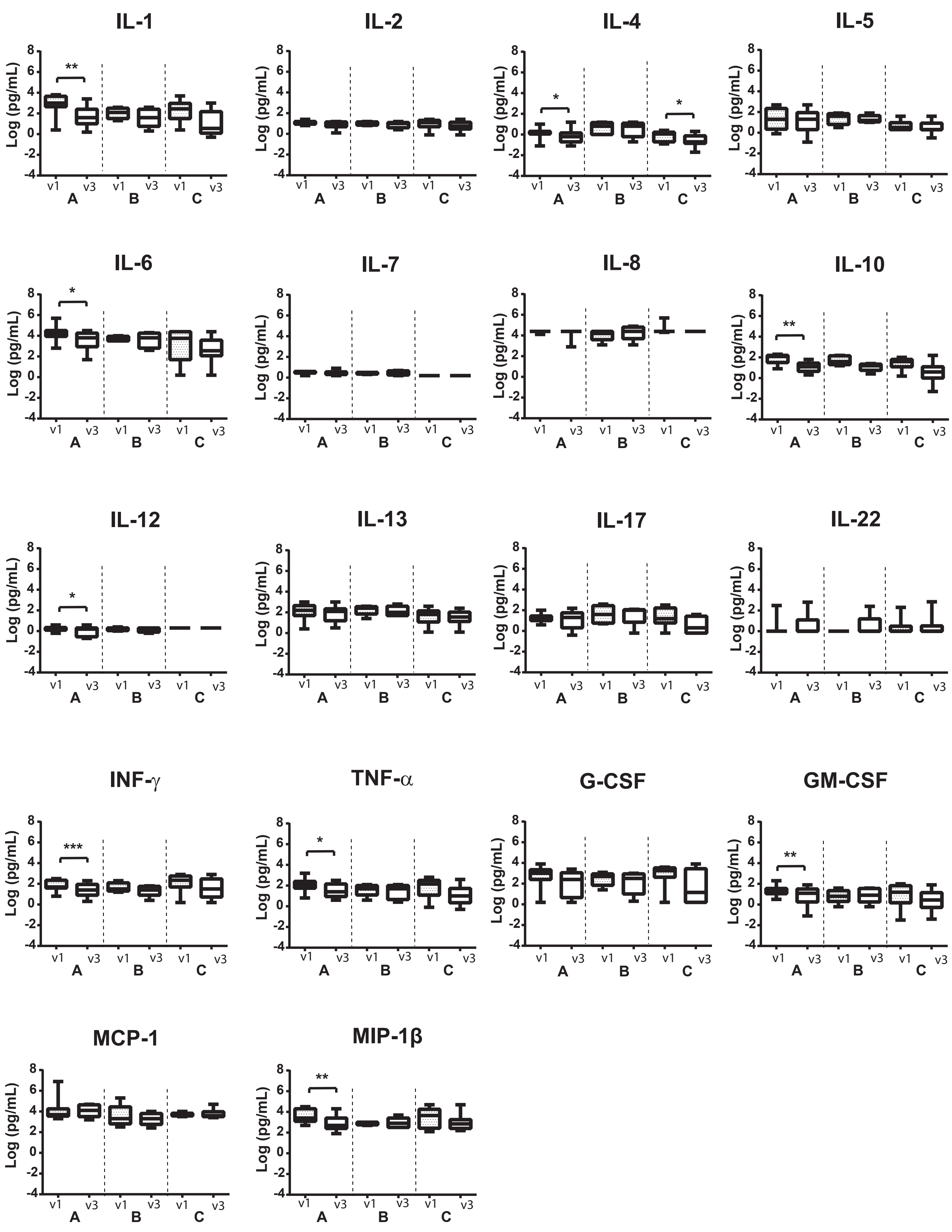
IL-1
IL-2
IL-4
IL-5
IL-6
IL-7
IL-8
IL-10
IL-12
IL-13
IL-17
IL-22
INF-γ
TNF-α
G-CSF
GM-CSF
MCP-1
MIP-1β
Log (pg/mL)
8
6
4
2
0
-2
-4
v1
v3
A
B
C
*
**
***

Supplemental Figure 3

F2

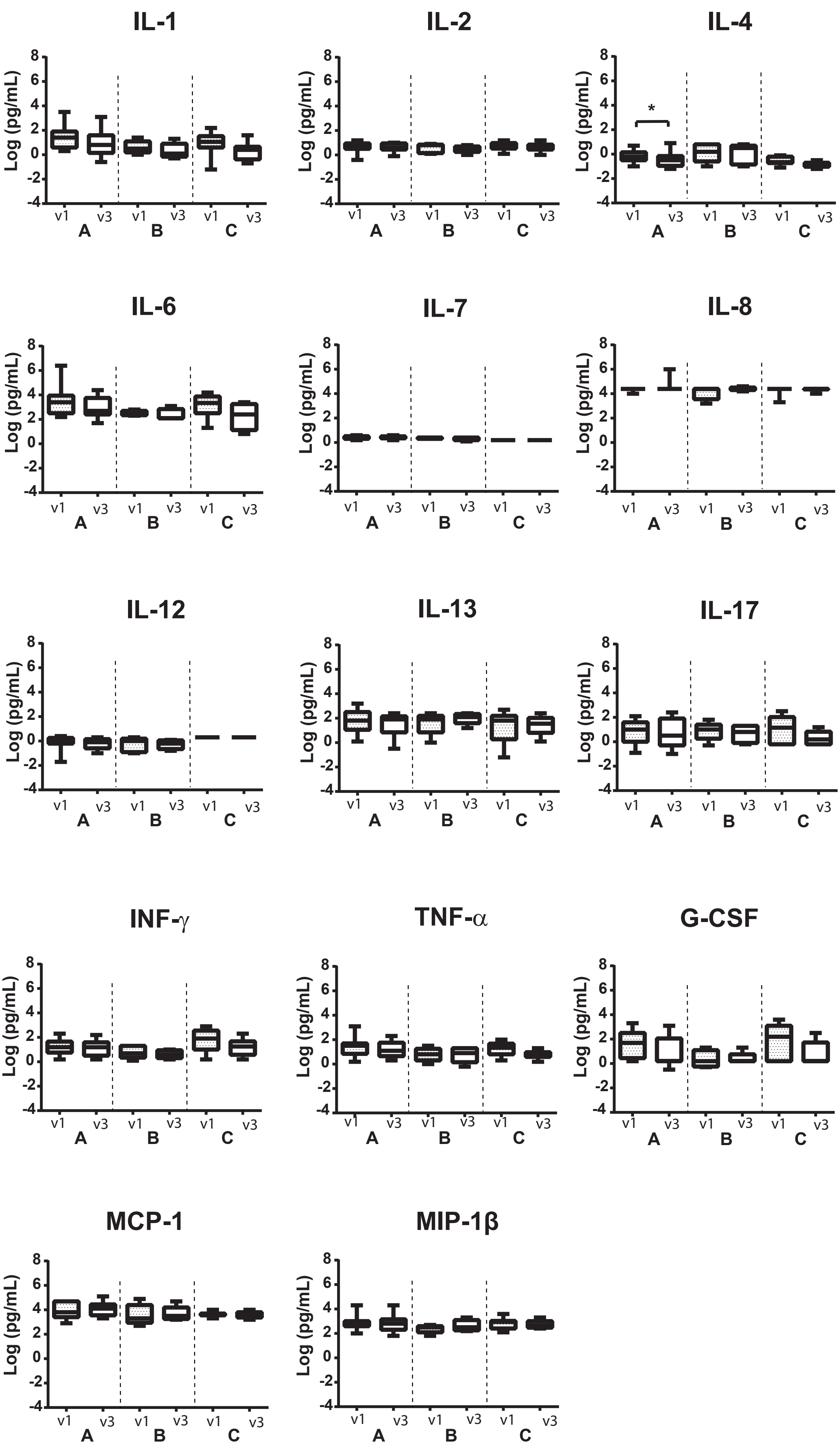

Supplemental Figure 4

F1+F2

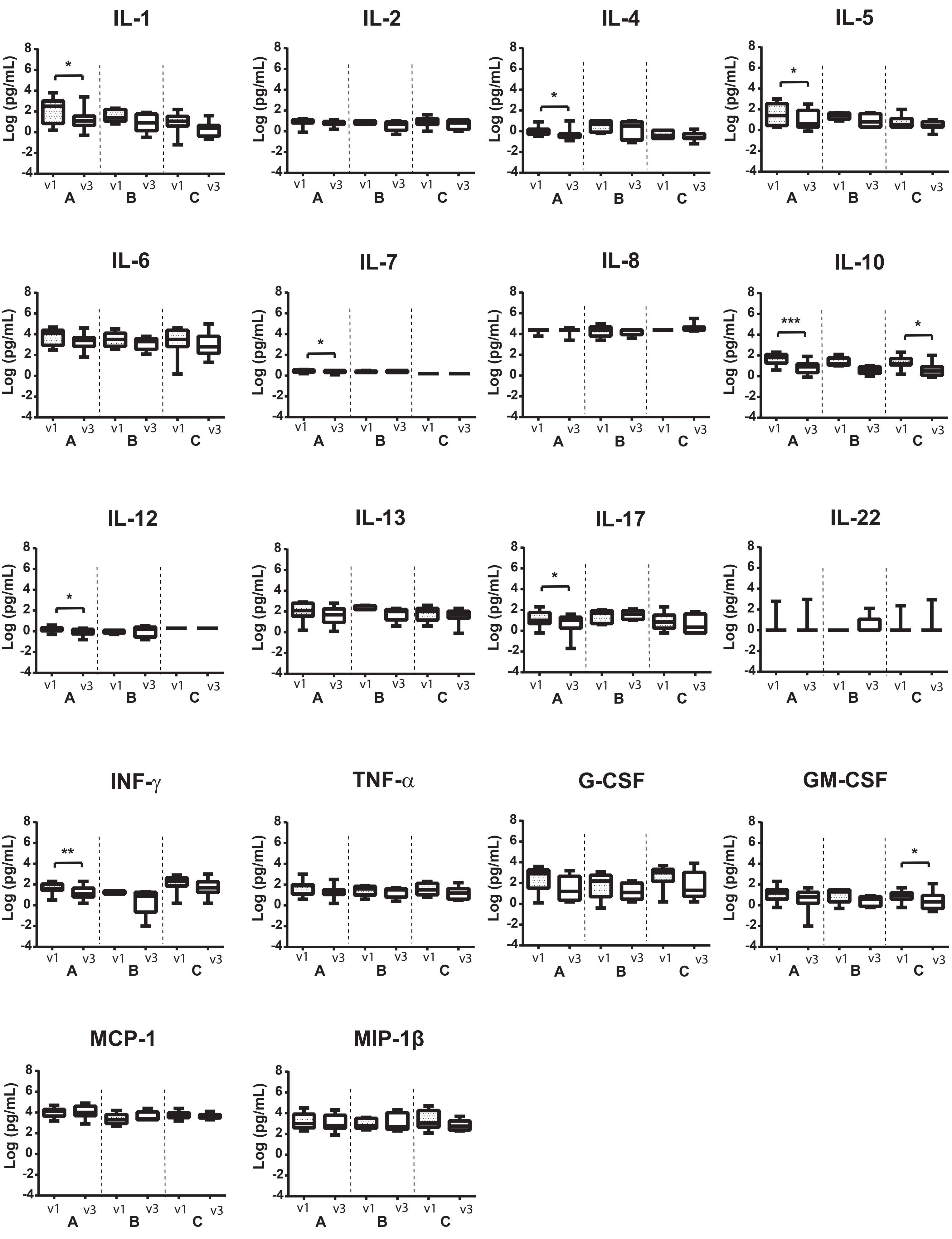

Supplement: Supplementary file 1 — Supplementary Information [file 41598_2017_10278_MOESM1_ESM.pdf]
